# Associations of Handgrip Strength and Testosterone With Cerebral White Matter Hyperintensity and Microstructural Injury

**DOI:** 10.1002/jcsm.13833

**Published:** 2025-06-04

**Authors:** Yuna Li, Shan Tian, Yuan Qiao, Chaohua Cong, Junting Yang, Shanshan Cao, Xirui Zhu, Lei Zhao, Panlong Li, Jingjing Su

**Affiliations:** ^1^ Department of Neurology, Shanghai Ninth People's Hospital Shanghai Jiao Tong University School of Medicine Shanghai China; ^2^ School of Electrical and Information Engineering Zhengzhou University of Light Industry Zhengzhou China; ^3^ Department of Medical Imaging Henan Provincial People's Hospital & Zhengzhou University People's Hospital Zhengzhou China

**Keywords:** cerebral small vessel disease, handgrip strength, testosterone, white matter hyperintensity, white matter microstructural injury

## Abstract

**Background:**

White matter hyperintensity (WMH) is one of the key imaging markers of cerebral small vessel disease (CSVD) and white matter microstructural injury may occur earlier than WMH. However, the associations of handgrip strength (HGS) and serum total testosterone (STT) with WMH and microstructural injury have not been thoroughly investigated. Therefore, we aimed to explore the associations of HGS and STT with WMH and microstructural injury, measured by fractional anisotropy (FA), mean diffusivity (MD), intracellular volume fraction (ICVF), and isotropic volume fraction (ISOVF).

**Methods:**

A total of 34 832 participants from the UK Biobank (aged 64.15 ± 7.75 years, 50.9% male) were included in the study. Cross‐sectional analyses were conducted using three linear regression models to assess the relationships between HGS, STT and neuroimaging outcomes. Sensitivity analyses were performed to validate the robustness of the linear regression results. Longitudinal, mediation and Mendelian randomization (MR) analyses were conducted to explore the longitudinal impact of HGS on WMH, the mediating effects of STT between HGS and neuroimaging outcomes, and probable causal relationships between HGS, STT and WMH.

**Results:**

A greater HGS was linearly associated with a smaller WMH volume (β = −0.006642, *p* < 0.001) and fuller microstructure (*p* < 0.001). These relationships persisted when stratified by sex or age decade and were supported by the results of the longitudinal analysis (β = −26.4, *p* = 0.0145). In females, STT was found to be linearly negatively related to WMH volume (β = −0.109, *p* < 0.001), MD and ISOVF, and linearly positively related to FA and ICVF (*p* < 0.001). In males, STT was linearly negatively linked to WMH volume (β = −0.016, *p* < 0.001) and MD (β = −1.6 $\times 10^{-7}$, *p* = 0.007), and positively associated to ICVF (β = 0.00012, *p* = 0.043). STT mediated the association between HGS and WMH volume (mediation proportion: 1.06% in males and 1.57% in females), and the possible causality was suggested for males through MR analysis: the positive causality between HGS and STT (*p* = 0.0394) and the negative causality between STT and WMH (*p* = 0.0787).

**Conclusions:**

Our study revealed that greater HGS was linearly associated with reduced WMH volume and less white matter microstructural injury, mediated by STT. Improving muscle function may contribute to deferring white matter damage and preventing stroke and dementia, offering a feasible secondary prevention measure for CSVD.

## 1 Introduction

White matter hyperintensity (WMH), one of the most common imaging markers of cerebral small vessel disease (CSVD), is the area of strong signal observed in the white matter on T2‐weighted and fluid attenuated inversion recovery (FLAIR) magnetic resonance imaging (MRI)^1^, increasing the risk for future stroke, dementia, and death [1, 2, 3]. Histopathologically, WMH corresponds to regions of demyelination, axonal loss, gliosis and parenchymal destruction [4, 5, 6]. However, white matter microstructural injury is not limited to visible WMH: it can also extend to regions that show no apparent abnormalities on FLAIR, known as normal‐appearing white matter (NAWM) in CSVD patients [7], which indicates that microstructural injury may occur earlier than WMH. Diffusion MRI (dMRI) can be employed to quantify the microstructural injury to white matter. Various dMRI metrics, such as fractional anisotropy (FA), mean diffusivity (MD), intracellular volume fraction (ICVF), and isotropic volume fraction (ISOVF), are used to assess the integrity of white matter microstructure and function. FA decreases and MD increases as white matter sustains damage [8]. ICVF reflects neurite density, and ISOVF reflects extracellular water diffusion [9]. Furthermore, lower FA and higher MD in the NAWM independently predict the conversion to WMH [10, 11]. Therefore, exploring the risk factors for WMH and microstructural injury could contribute to deferring white matter injury.

Muscle, an essential component of the human body accounting for approximately 40% of body weight, plays a crucial role in promoting blood circulation. Lower muscle strength is associated with worse health outcomes including cardiovascular disease, respiratory disease and cancer [12, 13]. Exercise and a protein‐rich diet can enhance muscle development and strength. Handgrip strength (HGS), a reliable measure of muscle strength, reflects overall vitality and physical function during aging [14]. HGS has been proposed to be a strong predictor of all‐cause or cause‐specific mortality, such as cardiovascular and respiratory diseases [15, 16], and is an indispensable biomarker for older adults [17]. Several studies have indicated a link between HGS and brain function, encompassing cognition, mobility and mental health, with most findings suggesting that greater HGS at baseline protects against declines in brain function [18, 19, 20]. In terms of brain structure, recent studies have demonstrated a correlation between increased HGS and reduced WMH [21], but the underlying mechanism has not been identified. Previous studies have shown that testosterone is positively associated with HGS [22] and serves as a significant moderator between vascular risk factors and WMH in males [23]. In addition, positive associations were observed between changes in testosterone and alterations in FA within the corpus callosum, cingulum cingulate and corticospinal tract in females [24]. Based on these findings, we hypothesize that HGS is associated with WMH and white matter microstructural injury, mediated by serum total testosterone (STT).

We aimed to analyse the associations of HGS and STT with both WMH volume and the extent of microstructural damage, to examine the mediating effect of STT between HGS and neuroimaging outcomes, to explore possible causality in cross‐sectional analysis and to investigate the longitudinal correlation between changes in HGS and WMH progression.

## 2 Methods

### 2.1 Study Population and Sample

The UK Biobank (UKB) is a prospective cohort study that consists of over 500 000 individuals from the United Kingdom, collecting data on e.g. population characteristics, lifestyle, medication and operation history, biological samples and imaging. In 2014 and 2019, baseline and first follow‐up MRI scans were undergone. Individuals with brain MRI data were included (*n* = 43 978) and without HGS or STT data were excluded (*n* = 7576) in our study. Besides, participants with diseases disturbing cerebral white matter assessment including multiple sclerosis or other demyelinating diseases, encephalitis, encephalomyelitis, intracranial abscess or granuloma, brain cancer, cerebral infarction or systemic lupus erythematosus (*n* = 632), and those in conditions affecting STT levels including disorders of pituitary gland, adrenogenital disorders, testicular dysfunction, testicular cancer, adrenal cancer, orchidectomy history, or the use of androgen, anti‐androgen, oestrogen, antioestrogen, progesterone or 5a‐reductase inhibitor medications (*n* = 855), were excluded. Prior to conducting analyses, we excluded outliers located outside the range of mean ±3 standard deviations for HGS (*n* = 83). Ultimately, we included a total of 34 832 participants in the cross‐sectional analysis. Three thousand five hundred seventy‐eight participants with follow‐up brain MRI data among them were included in the longitudinal analysis. On average, 2.60 years passed between two MRI scans. The UKB study was approved by the North West Multi‐Centre Research Ethics Committee, and all participants provided written informed consent. Our study also received approval from the UKB (application 94 885). Please refer to Table S1 for the field identifications, Table S2 for the timeline of the collected data and Figure S1 for the study process details.

### 2.2 Variables

#### 2.2.1 Exposures

Due to the unavailability of follow‐up STT data, we selected baseline STT and the second follow‐up HGS as exposures to correspond with initial brain MRI data from the UKB. Both left and right HGS were measured by a trained researcher using a Jamar J00105 hydraulic hand dynamometer following standardized procedures, and the average was calculated to characterize HGS. STT was assayed with a competitive binding chemiluminescent immunoassay.

#### 2.2.2 Neuroimaging Outcomes

We utilized neuroimaging data associated with white matter lesions from the UKB, including the total volume of WMH and microstructural injury indicators (FA, MD, ICVF and ISOVF). These imaging data were acquired by a standard Siemens Skyra 3 T scanner employing specific acquisition protocols, an image processing pipeline and derived measures. The primary outcome was WMH volume estimated from T2‐weighted FLAIR images. To ensure adherence to a Gaussian distribution, the WMH volume was logit‐transformed. The secondary outcomes included FA, MD, ICVF and ISOVF, which were calculated via dMRI with diffusion tensor imaging (DTI) fitting and neurite orientation dispersion and density imaging (NODDI) modelling. These dMRI parameters were separately derived as weighted means across 27 white matter tracts from the UKB. For each parameter, we computed the mean value of all 27 tracts as a global outcome for analysis.

#### 2.2.3 Covariates

To mitigate the influence of confounding factors, we incorporated the following covariates: sex, age at the time of the assessment, qualifications, smoking and alcohol consumption status, diabetes history, high blood pressure (HBP), frequency of physical activity, body mass index (BMI), high‐density lipoprotein cholesterol (HDL‐C) and low‐density lipoprotein (LDL) [25]. Qualifications were categorized into college or university degree, A levels/AS levels or equivalent, and lower [26]. HBP was defined as a mean systolic blood pressure over 140 mmHg or a mean diastolic blood pressure over 90 mmHg, or a diagnosis of HBP by a doctor or the use of blood pressure medication. The frequency of physical activity was quantified as the number of days with 10 min or more of moderate physical activity in a typical week.

### 2.3 Statistical Analyses

For missing covariates, we employed mean imputation for continuous variables and a missing indicator approach for categorical variables (all included covariates <20% missing).

#### 2.3.1 Cross‐Sectional Analysis

Descriptive analysis was conducted by sex. Three linear regression models were applied to examine the relationships between exposures (HGS and STT) and neuroimaging outcomes. Model 1 served as the base model without any covariate adjustment. Model 2 was adjusted for sex and age, as HGS and STT varied with sex and age. Model 3 was adjusted for all included covariates. To assess the appropriateness of the linear models, diagnostic plots of residuals were examined for goodness of fit.

#### 2.3.2 Sensitivity Analysis

Considering the sex differences in HGS and STT, all models were stratified by sex. Moreover, for physiological brain aging, an additional analysis was conducted based on age group (<60, 60–70 and ≥70 years) in model 3. This analysis was adjusted for all included covariates within each age group, including age, sex, and so on. Due to the unreliability of competitive binding chemiluminescent immunoassay for STT measurement, those with low STT levels (<10.4 nmol/L) were excluded from the analysis about the relationship between STT and neuroimaging outcomes. White participants occupy the majority of our study sample (96.7%). In view of the effect of ethnicity on HGS [27, 28], we only included White participants (*n* = 33 696) and reran the cross‐sectional analysis.

#### 2.3.3 Longitudinal Analysis

To evaluate the longitudinal impact of HGS on WMH, three linear regression models were applied to examine the correlation between HGS changes and WMH progression, which were defined as the difference in values between two MRI scans. In addition, we made comparisons about the characteristics of the population between the cross‐sectional and longitudinal analyses.

#### 2.3.4 Mediation Analysis

The mediating effects of STT on the associations between HGS and neuroimaging outcomes were assessed, and this analysis was stratified by sex. The extents of mediation were expressed as percentages.

#### 2.3.5 Mendelian Randomization (MR) Analysis

Data were extracted from the IEU Open Genome‐Wide Association Study (GWAS) Project (https://gwas.mrcieu.ac.uk/). The largest publicly available GWAS focused on the European cohort was selected for HGS, STT and WMH volume. The basic information of the GWAS statistics is shown in Table S3.

Relevant genetic variables were selected as instrumental variables. To reduce the bias arising from weakly correlated instrumental variables, only SNPs substantially associated (*p* < 5 × 10^−8^) with HGS or STT were considered. Additionally, to ensure the independence of instrument variables, we excluded SNPs with linkage disequilibrium (LD) values less than 0.7. The LD was estimated based on the 1000 Genomes Project. To minimize the impact of pseudovariation, SNPs with a minor allele frequency less than 0.01 were also excluded. Ambiguous SNPs with incompatible alleles (e.g. A/G vs. A/C) were excluded. Palindromic SNPs with ambiguous alleles (e.g. A/T vs. G/C), incorrect effect alleles (e.g. G/T vs. T/G) or strand issues (e.g. G/T vs. C/A) were harmonized by flipping the outcome variants.

To examine whether the associations were likely causal, inverse‐variance weighted (IVW) two‐sample MR analysis was applied as the main analysis to estimate the effect of HGS on STT and of STT on WMH volume only in males. The IVW method provides an unbiased effect in the absence of horizontal pleiotropy (when the genetic variants are associated with the outcome through pathways other than the exposure pathway) or when horizontal pleiotropy is balanced. In addition, we performed other MR analyses, including MR‐Egger, weighted median, simple mode and weighted mode analyses as sensitivity analyses. Directional pleiotropy was assessed based on the significant difference in the intercept from zero (*p* < 0.05) in MR‐Egger regression. A “leave‐one‐out” sensitivity analysis was performed to evaluate whether the analysis was biased by a single SNP with a particularly large horizontal pleiotropic effect. Funnel plots were also drawn to assess the heterogeneity among SNPs. Considering the sex difference in STT, we stratified the MR analysis between STT and WMH volume by sex. However, only GWAS statistics for males were selected due to the lack of female‐specific data.

A significance level of *p* < 0.05 was considered to indicate statistical significance. All the statistical analyses and figure preparations were conducted with R software version 4.2.3.

## 3 Results

### 3.1 Baseline Characteristics

The baseline characteristics of the 34 832 participants (aged 64.15 ± 7.75 years, 50.9% male) included in the cross‐sectional study are presented by sex in Table 1, which shows the means with standard deviations for continuous variables and the numbers with percentages for categorical variables. Males tended to be older, smokers and alcohol drinkers and had stronger HGS, higher STT levels, no A/AS levels or above, less activity, greater BMIs, HBP disease, diabetes, lower HDL‐C and LDL levels (*p* < 0.001).

**TABLE 1:** Characteristics of 34 832 UK Biobank participants according to gender group.

Characteristics	Overall	Male	Female	*p*
**Number**	34 832	17 739 (50.9%)	17 093 (49.1%)	—
**Handgrip strength (kg)**	30.30 (10.21)	37.44 (8.24)	22.89 (5.84)	< 0.001
**Testosterone (nmol/L)**	6.77 (6.12)	12.23 (3.54)	1.11 (0.60)	< 0.001
**Age (years)**	64.15 (7.75)	64.97 (7.83)	63.31 (7.58)	< 0.001
**Qualifications**				< 0.001
Unknown	327 (0.9%)	163 (0.9%)	164 (1.0%)	
None	13 437 (38.6%)	6853 (38.6%)	6584 (38.5%)	
College/university	16 860 (48.4%)	8746 (49.3%)	8114 (47.5%)	
A/AS levels	4208 (12.1%)	1977 (11.1)	2231 (13.1%)	
**Smoking**	12 836 (36.9%)	7179 (40.5%)	5657 (33.1%)	< 0.001
**Alcohol**	33 558 (96.3%)	17 243 (97.2%)	16 315 (95.4%)	< 0.001
**Activity (days)**	4.03 (2.17)	3.96 (2.16)	4.09 (2.19)	< 0.001
**HBP**	16 574 (47.6%)	9732 (54.9%)	6842 (40.0%)	< 0.001
**BMI (kg/m** ^**2**^ **)**	26.60 (4.35)	26.94 (3.86)	26.24 (4.79)	< 0.001
**Diabetes**	1827 (5.2%)	1223 (6.9%)	604 (3.5%)	< 0.001
**HDL‐C (mmol/L)**	1.46 (0.35)	1.32 (0.29)	1.61 (0.35)	< 0.001
**LDL (mmol/L)**	3.58 (0.83)	3.57 (0.83)	3.60 (0.83)	< 0.001

Abbreviations: BMI, body mass index; HBP, high blood pressure; HDL‐C, high‐density lipoprotein cholesterol; LDL, low‐density lipoprotein.

### 3.2 HGS, STT and Neuroimaging Outcomes in All Participants

Table 2 presents the significant linear relationships between HGS and neuroimaging outcomes across all models. Specifically, WMH volume decreased with increasing HGS (β = −0.006642, *p* < 0.001). In terms of the dMRI parameters, FA and ICVF increased with increasing HGS, while MD and ISOVF decreased. As shown in Table 3, the *p* values of some linear regression models were less than 0.05, but the diagnostic plots of residuals showed poor linear goodness of fit. Therefore, there were no linear relationships between STT and WMH volume or microstructural injury indicators in the entire cross‐sectional analysis cohort.

**TABLE 2:** Associations between handgrip strength and neuroimaging outcomes among all participants.

	Model 1		Model 2		Model 3	
HGS	β (95%CI)	*p*	β (95%CI)	*p*	β (95%CI)	*p*
**log_WMH**	−0.0066420 (−0.007672396, −0.005611577)	< 0.001	−0.0056107 (−0.006944655, −0.004276705)	< 0.001	−0.0054438 (−0.0067636762, −0.004123958)	< 0.001
**FA**	2.657e‐04 (0.0002501417, 0.0002813034)	< 0.001	1.178e‐04 (0.0000947506, 0.0001407410)	< 0.001	1.121e‐04 (8.908622e‐05, 1.351537e‐04)	< 0.001
**MD**	−1.581e‐07 (−1.855320e‐07, −1.307195e‐07)	< 0.001	−1.827e‐07 (−2.207676e‐07, −1.446063e‐07)	< 0.001	−1.765e‐07 (−2.146088e‐07, −1.384289e‐07)	< 0.001
**ICVF**	1.811e‐04 (0.0001538444, 0.0002083874)	< 0.001	7.671e‐05 (3.686527e‐05, 0.0001165556)	< 0.001	7.031e‐05 (0.0000303806, 0.0001102469)	< 0.001
**ISOVF**	−2.835e‐05 (−3.915935e‐05, −1.754888e‐05)	< 0.001	−7.939e‐05 (−9.482408e‐05, −6.396023e‐05)	< 0.001	−7.819e‐05 (−9.366350e‐05, −6.271869e‐05)	< 0.001

*Note:* Model 1 was not adjusted. Model 2 was adjusted for sex and age. Model 3 was adjusted for sex, age, qualifications, smoking status, alcohol drinking status, diabetes history, HBP, frequency of physical activities, BMI, HDL‐C, LDL.

Abbreviations: FA, fractional anisotropy; HGS, hand grip strength; ICVF, intracellular volume fraction; ISOVF, isotropic volume fraction; log_WMH, logit‐transformed value of white matter hyperintensity volume; MD, mean diffusivity.

**TABLE 3:** Associations between serum total testosterone and neuroimaging outcomes among all participants.

	Model 1		Model 2		Model 3	
Testosterone	β (95%CI)	*p*	β (95%CI)	*p*	β (95%CI)	*p*
**log_WMH**	0.0125173 (0.01078618, 0.01424851)	< 0.001	−0.0097049 (−0.01332906, −0.00608072)	< 0.001	−0.0008147 (−0.0044429039, 0.002813543)	0.659864
**FA**	2.985e‐04 (0.0002720686, 0.0003249191)	< 0.001	2.418e‐05 (−3.832611e‐05, 0.0000866877)	0.448	−3.097e‐06 (−6.641527e‐05, 6.022034e‐05)	0.923614
**MD**	2.938e‐07 (2.477245e‐07, 0.0000003398)	< 0.001	−2.143e‐08 (−1.249053e‐07, 8.204952e‐08)	0.684834	−1.328e‐08 (−1.179597e‐07, 9.139608e‐08)	0.803599
**ICVF**	7.360e‐06 (−3.858669e‐05, 5.330634e‐05)	0.754	3.624e‐05 (−7.193941e‐05, 0.0001444152)	0.511	6.003e‐05 (−4.961606e‐05, 0.0001696826)	0.283224
**ISOVF**	1.601e‐04 (0.0001420251, 0.0001782032)	< 0.001	9.706e‐06 (−3.223397e‐05, 5.164678e‐05)	0.65	3.454e‐05 (−7.995577e‐06, 7.706633e‐05)	0.111493

Model 1 was not adjusted. Model 2 was adjusted for sex and age. Model 3 was adjusted for sex, age, qualifications, smoking status, alcohol drinking status, diabetes history, HBP, frequency of physical activities, BMI, HDL‐C, LDL.

Abbreviations: FA, fractional anisotropy; ICVF, intracellular volume fraction; ISOVF, isotropic volume fraction; log_WMH, logit‐transformed value of white matter hyperintensity volume; MD, mean diffusivity.

### 3.3 Sensitivity Analysis

**FIGURE 1:**
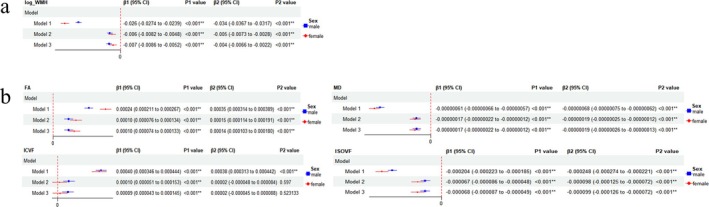
Associations between handgrip strength and neuroimaging outcomes stratified by sex. (a) Associations between handgrip strength and white matter hyperintensity (WMH) stratified by sex. (b) Associations between handgrip strength and white matter microstructural injury stratified by sex, FA, fractional anisotropy; MD, mean diffusivity; ICVF, intracellular volume fraction; ISOVF, isotropic volume fraction. (Model 1 was not adjusted. Model 2 was adjusted for sex and age. Model 3 was adjusted for sex, age, qualifications, smoking status, alcohol drinking status, diabetes history, HBP, frequency of physical activities, BMI, HDL‐C, LDL.)

**FIGURE 2:**
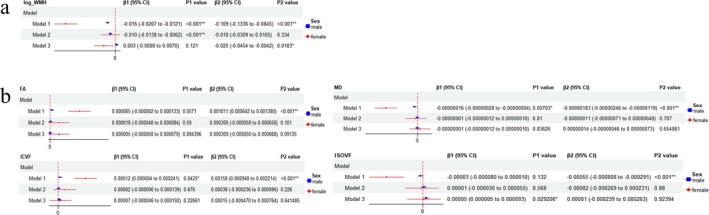
Associations between serum total testosterone and neuroimaging outcomes stratified by sex. (a) Associations between serum total testosterone and white matter hyperintensity (WMH) stratified by sex. (b) Associations between serum total testosterone and white matter microstructural injury stratified by sex, FA, fractional anisotropy; MD, mean diffusivity; ICVF, intracellular volume fraction; ISOVF, isotropic volume fraction. (Model 1 was not adjusted. Model 2 was adjusted for sex and age. Model 3 was adjusted for sex, age, qualifications, smoking status, alcohol drinking status, diabetes history, HBP, frequency of physical activities, BMI, HDL‐C, LDL.)

HGS exhibited negative linear correlations with WMH volume (β = −0.026, *p* < 0.001 in males; β = −0.034, *p* < 0.001 in females), MD and ISOVF, but it exhibited positive linear correlations with FA and ICVF regardless of sex (Figure 1). STT was found to be linearly negatively related to WMH volume (β = −0.109, *p* < 0.001), MD and ISOVF, and linearly positively related to FA and ICVF in females, while in males, it was only linearly negatively linked to WMH volume (β = −0.016, *p* < 0.001) and MD, and linearly positively associated to ICVF. The *p* value of the linear regression between STT and ISOVF in males was also less than 0.05, but the diagnostic plots of residuals showed poor linear goodness of fit (Figure 2). After stratification by age and adjustment for all included covariates, the associations of HGS with WMH and microstructural injury were still significant across three age groups (Figure S2), but the relationships between STT and neuroimaging outcomes (FA and ICVF) were found to be significant only within the age group of individuals aged 70 years or older (Figure S3). In addition, STT was also found to be linearly negatively associated with WMH volume among participants with a STT concentration ≥10.4 nmol/L (Table S4). Among White participants, the outcomes were in keeping with the main analysis in all participants (Table S5, Table S6).

### 3.4 Longitudinal Analysis

**FIGURE 3:**
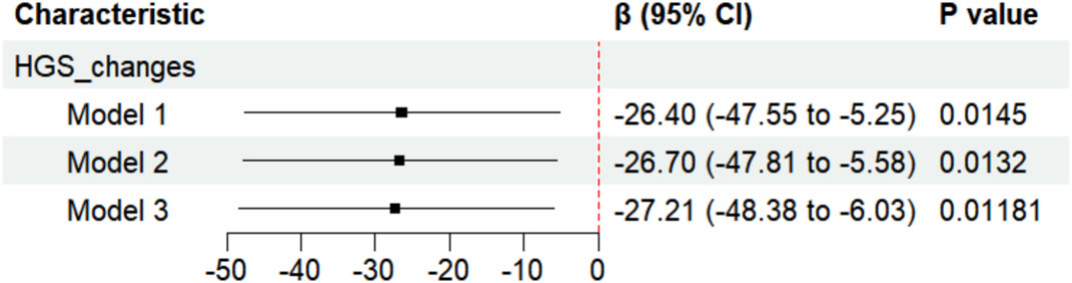
Associations of handgrip strength changes with white matter hyperintensity progression between two scans. Model 1 was not adjusted. Model 2 was adjusted for sex and age. Model 3 was adjusted for sex, age, qualifications, smoking status, alcohol drinking status, diabetes history, HBP, frequency of physical activities, BMI, HDL‐C, LDL. Abbreviations: HGS, handgrip strength changes.

HGS changes were linearly related to WMH progression (β = −26.4, *p* = 0.0145) between two scans, and the negative association was consistent across three models (Figure 3). The characteristics of the population in the cross‐sectional and longitudinal analyses are shown in Table S7.

### 3.5 Mediation Analysis

In males, STT level mediated 1.06% of the association between greater HGS and decreased WMH volume, while it had no mediating effect on the relationship between greater HGS and decreased MD or increased ICVF. In females, STT level played a mediating role in the associations between HGS and all neuroimaging outcomes. Specifically, STT mediated 1.57%, 1.41%, 1.26%, 2.12% and 0.98% of the associations of greater HGS with decreased WMH volume, increased FA, decreased MD, increased ICVF and decreased ISOVF, respectively (Figure S4, Figure S5).

### 3.6 MR Analysis

For HGS, the IVW analysis yielded evidence supporting a positive causal association between HGS and STT (β = 0.1919; *p* = 0.0394). This causal relationship remained consistent for other MR analyses. Furthermore, MR‐Egger regression analysis revealed no evidence of directional pleiotropy (intercept = −0.002898, *p* = 0.4656). The leave‐one‐out analysis did not reveal any single SNP that strongly influenced the overall effect of HGS on STT. Moreover, the symmetry observed in the funnel plots indicated no violations of the instrumental variable (IV) assumptions (Figure S6).

For STT, the IVW analysis provided no evidence to support a significant causal association between STT and WMH volume (β = −0.2144; *p* = 0.0787), but the results suggested that those with higher STT levels tended to show lower WMH volumes. A negative causal relationship between STT and WMH volume was found in the weighted median (β = −0.3448; *p* = 0.0059), simple mode methods (β = −0.9046; *p* = 0.0026) and weighted mode (β = −0.5101; *p* = 0.0009) analyses for males. These outcomes were unaffected by directional pleiotropy in the MR‐Egger regression analysis (intercept = 0.0000545, *p* = 0.9946). Furthermore, the leave‐one‐out analysis and funnel plots supported the absence of IV assumption violations (Figure S7).

## 4 Discussion

To our knowledge, this is the first large prospective cohort study to thoroughly investigate the associations of HGS and STT with WMH and microstructural injury while exploring the mediating effect of STT and the causal relationship therein. Our findings indicated a linear association of HGS with both WMH volume and all measures of microstructural injury. These associations remained significant after stratification by sex and age. Furthermore, longitudinal analysis revealed that HGS changes were linearly negatively related to WMH progression. A linear negative correlation between STT and WMH volume was confirmed regardless of sex; however, the correlations of STT with microstructural injury indicators varied by sex and age decade. In males, the linear associations of STT with MD and ICVF were confirmed, while in females, STT was linearly linked with all neuroimaging outcomes. STT was linearly associated with FA and ICVF adjusted for all included covariates only within the ≥70 years age group. Moreover, we confirmed the mediating effects of STT between HGS and neuroimaging outcomes, which could be explained by the possible causal effect of HGS on STT and of STT on WMH volume. Nevertheless, the mediating effects were small and the most likely reason for this is the existence of other moderating factors. These potential moderating factors may interfere with the relationship between STT and WMH, resulting in a decrease in mediating effect.

The current results on the relationship between HGS and WMH volume are in line with previous findings. Sachdev et al. reported that HGS was negatively associated with WMH in various brain regions [29, 30]. Kilgour et al. confirmed that a greater accumulation of WMH was associated with reduced grip strength [31]. Zhang et al. also reported that HGS was negatively correlated with WMH grade evaluated by the Fazekas visual grade scale [32]. In our study, we found that HGS was negatively and linearly associated with WMH volume which is consistent with the findings of another UKB cohort study that concluded that a 5‐kg decrease in HGS was linked to greater WMH volume [21]. To date, few studies have demonstrated the relationship between HGS and WMH volume from a longitudinal perspective. In our longitudinal analysis, we innovatively confirmed that HGS changes were linearly negatively related to WMH progression. To date, no previous studies have demonstrated the connection between HGS and white matter microstructural injury. We first found that HGS was negatively associated with MD and ISOVF and positively related to FA and ICVF, which suggested that greater HGS may be protective for white matter integrity. Notably, the associations of HGS with cerebral white matter injury were independent of sex. Additionally, the associations were consistent in three age groups (<60, 60–70 and ≥70 years). Based on our findings, we hypothesized that better muscle function or greater muscle strength could delay white matter injury. One of the underlying mechanisms may be that muscle produces and secretes hundreds of myokines involved in neuronal differentiation, plasticity, cell survival and regulating brain functions, which indicates the existence of muscle‐brain cross‐talk [33, 34]. The common cause hypothesis proposed by Lindenberger and Baltes [35] postulates that there are shared underlying processes that drive aging throughout the human body. This also explains why associations between muscle function and brain structure or function are reasonable.

For STT, an Atherosclerosis Risk in Communities (ARIC) study showed that testosterone was not associated with the percentage of WMH in community‐dwelling men after adjustment [25]. The discrepancy between their results and ours might be attributed to the long lag between testosterone measurement and brain imaging in our study. Alqarni et al. also reported no significant associations of testosterone with total WMH, periventricular WMH and deep WMH in men [23], which was not consistent with our findings in males. Nevertheless, several limitations were that those with diseases disturbing white matter assessment and conditions affecting STT levels were not excluded from their study population. Our results suggested that higher STT levels were related to less WMH volume regardless of sex, which could be attributed to the neuroprotection and myelin regeneration of testosterone [36, 37]. Perrin et al. reported that white matter growth shows striking sexual dimorphism during adolescence when testosterone levels rise rapidly [38]. In addition, previous studies have provided evidence to support the notion that women tend to have a greater load and faster progression of WMH with aging [29, 39]. Regarding the association between STT and white matter microstructural injury, Herting et al. reported that boys exhibited greater FA and lower MD than girls during adolescence [40], which aligns with our findings. In addition, Ho et al. also found that changes in testosterone were positively related to changes in FA within the corpus callosum, cingulum cingulate and corticospinal tract in girls [24], supporting our results. However, the relationships between STT and other microstructural injury indicators have not been clarified. Our results extended these prior studies by considering sex and age as stratification factors, revealing that the association between STT and white matter injury was moderated by sex and age.

We discovered that both HGS and STT were directly related to WMH, and a previous study indicated that HGS was positively related to STT level [41]. These findings led us to propose that STT mediates the association between HGS and white matter injury. Strikingly, we confirmed the mediating role of STT in the relationship between HGS and neuroimaging outcomes, although the mediation effect was small. Furthermore, we performed the first MR analysis between HGS and STT, as well as between STT and WMH volume, suggesting possible causalities. Therefore, better muscle function may protect white matter integrity through increased STT, which provided us with insight into the mechanism by which muscle function protects white matter. Exercise (especially resistance training) and a nutritional diet rich in protein contributing to improving muscle function could delay white matter damage [42], and subsequently prevent the occurrence of stroke and dementia, which is particularly important for CSVD patients who mostly exhibit WMH.

## 5 Strengths and Limitations

The major strengths of our study include the use of a large, well‐characterized and prospective cohort consisting of adults from the United Kingdom. Additionally, we pioneered the examination of the mediating effect of STT on the relationship between HGS and WMH volume and the longitudinal influence of HGS on the WMH volume, establishing possible causal relationships among HGS, STT and WMH volume. However, there are several potential limitations in our current study. First, WMH volume was assessed in the whole brain rather than in specific regions. Second, the means of the left and right HGS was calculated and handedness was not taken into account. Third, we chose STT at baseline, which resulted in a time interval from the measurement of HGS and WMH volume (average 9.33 years). In the UKB, STT was assayed using a competitive binding chemiluminescent immunoassay, which may not be a reliable measurement. Fourth, although the current study was extensively adjusted for confounding factors and we conducted various sensitivity analyses to validate the robustness of the results, certain potential covariates were not adjusted for, which might partially exaggerate the observed effects. Fifth, the follow‐up period (average 2.6 years) between two scans in our study might be too short to capture significant changes in brain imaging. Sixth, only GWAS statistics for males were selected due to the lack of female‐specific data, which resulted in an unclear causal link between STT and WMH volume in females. Future studies should aim to replicate our findings in a more evenly ethnically distributed population, including people without white matter injuries, across longer follow‐up periods of MRI scans.

## 6 Conclusions

To summarize, greater HGS and STT were found to be significantly linearly associated with reduced WMH volume and less microstructural injury. The longitudinal analysis between HGS changes and WMH progression further clarified the association of HGS with WMH. STT mediated the relationships, and possible causalities were suggested. Improving overall vitality and muscle function could contribute to deferring white matter damage and preventing stroke and dementia, which are secondary prevention measures for CSVD.

## Ethics Statement

We declare that the UKB study was approved by the North West Multi‐Centre Research Ethics Committee, and all participants provided written informed consent.

## Conflicts of Interest

The authors declare no conflicts of interest.

## Supporting information


**TABLE S1** Field IDs used in analyses.
TABLE S2 The timeline of collected data.
FIGURE S1 Flowchart of the study process.
TABLE S3 The basic information of genome‐wide association study (GWAS) statistics.
FIGURE S2 Associations between handgrip strength and neuroimaging outcomes stratified by age decade.
FIGURE S3 Associations between serum total testosterone and neuroimaging outcomes stratified by age decade.
TABLE S4 Associations between serum total testosterone and neuroimaging outcomes among participants at ≥10.4 nmol/L serum total testosterone.
TABLE S5 Associations between handgrip strength and neuroimaging outcomes in the White.
TABLE S6 Associations between serum total testosterone and neuroimaging outcomes in the White.
TABLE S7 Characteristics of population in the cross‐sectional and longitudinal analyses.
FIGURE S4 The mediating effects of serum total testosterone between handgrip strength and white matter hyperintensity.
FIGURE S5 The mediating effects of serum total testosterone between handgrip strength and white matter microstructural injury in female.
FIGURE S6 Causal effects of handgrip strength (HGS) on serum total testosterone (STT).
FIGURE S7 Causal effects of serum total testosterone (STT) on white matter hyperintensity (WMH).
