# Supplementary material for: Associations of Handgrip Strength and Testosterone With Cerebral White Matter Hyperintensity and Microstructural Injury
Source: J Cachexia Sarcopenia Muscle. 2025 Jun 4;16(3):e13833. doi: 10.1002/jcsm.13833 (PMC12134939; doi:10.1002/jcsm.13833)
Supplement: Supplementary file 1 — TABLE S1 Field IDs used in analyses. TABLE S2 The timeline of collected data. FIGURE S1 Flowchart of the study process. TABLE S3 The basic information of genome‐wide association study (GWAS) statistics. FIGURE S2 Associations between handgrip strength and neuroimaging outcomes stratified by age decade. FIGURE S3 Associations between serum total testosterone and neuroimaging outcomes stratified by age decade. TABLE S4 Associations between serum total testosterone and neuroimaging outcomes among participants at ≥10.4 nmol/L serum total testosterone. TABLE S5 Associations between handgrip strength and neuroimaging outcomes in the White. TABLE S6 Associations between serum total testosterone and neuroimaging outcomes in the White. TABLE S7 Characteristics of population in the cross‐sectional and longitudinal analyses. FIGURE S4 The mediating effects of serum total testosterone between handgrip strength and white matter hyperintensity. FIGURE S5 The mediating effects of serum total testosterone between handgrip strength and white matter microstructural injury in female. FIGURE S6 Causal effects of handgrip strength (HGS) on serum total testosterone (STT). FIGURE S7 Causal effects of serum total testosterone (STT) on white matter hyperintensity (WMH). [file JCSM-16-e13833-s001.docx]

**Supplement**

**Table S1.** Field IDs used in analyses.

**Table S2.** The timeline of collected data.

**Figure S1.** Flow chart of the study process.

**Table S3.** The basic information of genome-wide association study (GWAS) statistics.

**Figure S2.** Associations between handgrip strength and neuroimaging outcomes stratified by age decade.

**Figure S3.** Associations between serum total testosterone and neuroimaging outcomes stratified by age decade.

**Table S4.** Associations between serum total testosterone and neuroimaging outcomes among participants at ≥10.4nmol/L serum total testosterone.

**Table S5.** Associations between handgrip strength and neuroimaging outcomes in the White.

**Table S6.** Associations between serum total testosterone and neuroimaging outcomes in the White.

**Table S7.** Characteristics of population in the cross-sectional and longitudinal analyses.

**Figure S4.** The mediating effects of serum total testosterone between handgrip strength and white matter hyperintensity.

**Figure S5.** The mediating effects of serum total testosterone between handgrip strength and white matter microstructural injury in female.

**Figure S6.** Causal effects of handgrip strength (HGS) on serum total testosterone (STT).

**Figure S7.** Causal effects of serum total testosterone (STT) on white matter hyperintensity (WMH).

**Table S1. Field IDs used in analyses.**

|  | **Items** | **Field ID** |
| --- | --- | --- |
| **Independent variable** | Hand grip strength (left) | 46 |
|  | Hand grip strength (right) | 47 |
|  | Testosterone | 30850 |
| **Covariate** | Sex | 31 |
|  | Age when attended assessment centre | 21003 |
|  | Qualifications | 6138 |
|  | Smoking status | 20116 |
|  | Alcohol drinker status | 20117 |
|  | Number of days/week of moderate physical activity 10+ minutes | 884 |
|  | Body mass index | 21001 |
|  | Diabetes | 2443 |
|  | [High density lipoprotein cholesterol](https://biobank.ndph.ox.ac.uk/showcase/field.cgi?id=30760) | 30760 |
|  | Low density lipoprotein | 30780 |
|  | Medication | 6177 |
|  | Diastolic blood pressure, automated reading | 4079 |
|  | Systolic blood pressure, automated reading | 4080 |
|  | Vascular/heart problems diagnosed by doctor | 6150 |
| **MRI markers** | White matter hyperintensity | 25781 |
|  | Fractional anisotropy | 25488-25514 |
|  | Mean diffusivity | 25515-25541 |
|  | Intracellular volume fraction | 25650-25676 |
|  | Isotropic volume fraction | 25704--25730 |
| **Confounding conditions** | other acute disseminated demyelination | 131044 |
|  | other demyelinating diseases of central nervous system | 131046 |
|  | cerebral infarction | 131366 |
|  | encephalitis, myelitis and encephalomyelitis | 131000 |
|  | encephalitis, myelitis and encephalomyelitis in diseases classified elsewhere | 131002 |
|  | intracranial and intraspinal abscess and granuloma | 131004 |
|  | intracranial and intraspinal abscess and granuloma in diseases classified elsewhere | 131006 |
|  | systemic lupus erythematosus | 131894 |
|  | Cancer code | 20001 |
|  | Operation code | 20004 |
|  | hyperfunction of pituitary gland | 130724 |
|  | hypofunction and other disorders of pituitary gland | 130726 |
|  | adrenogenital disorders | 130730 |
|  | testicular dysfunction | 130738 |
|  | Treatment/medication code | 20003 |

**Table S2.** The timeline of collected data.

| **Time** | **Items** |
| --- | --- |
| **Initial assessment visit**  **(2006-2010)** | Testosterone  Sex  High density lipoprotein cholesterol  Low density lipoprotein  Vascular/heart problems diagnosed by doctor  other acute disseminated demyelination  other demyelinating diseases of central nervous system  cerebral infarction  encephalitis, myelitis and encephalomyelitis  encephalitis, myelitis and encephalomyelitis in diseases classified elsewhere  intracranial and intraspinal abscess and granuloma  intracranial and intraspinal abscess and granuloma in diseases classified elsewhere  systemic lupus erythematosus  hyperfunction of pituitary gland  hypofunction and other disorders of pituitary gland  adrenogenital disorders  testicular dysfunction |
| **Imaging visit (2014+)** | Hand grip strength (left)  Hand grip strength (right)  Age when attended assessment centre  Qualifications  Smoking status  Alcohol drinker status  Number of days/week of moderate physical activity 10+ minutes  Body mass index  Diabetes  Medication  Diastolic blood pressure, automated reading  Systolic blood pressure, automated reading  White matter hyperintensity  Fractional anisotropy  Mean diffusivity  Intracellular volume fraction  Isotropic volume fraction  Cancer code  Treatment/medication code  Operation code |
| **First repeat imaging visit (2019+)** | Hand grip strength (left)  Hand grip strength (right)  White matter hyperintensity |

**Figure S1. Flow chart of the study process.**


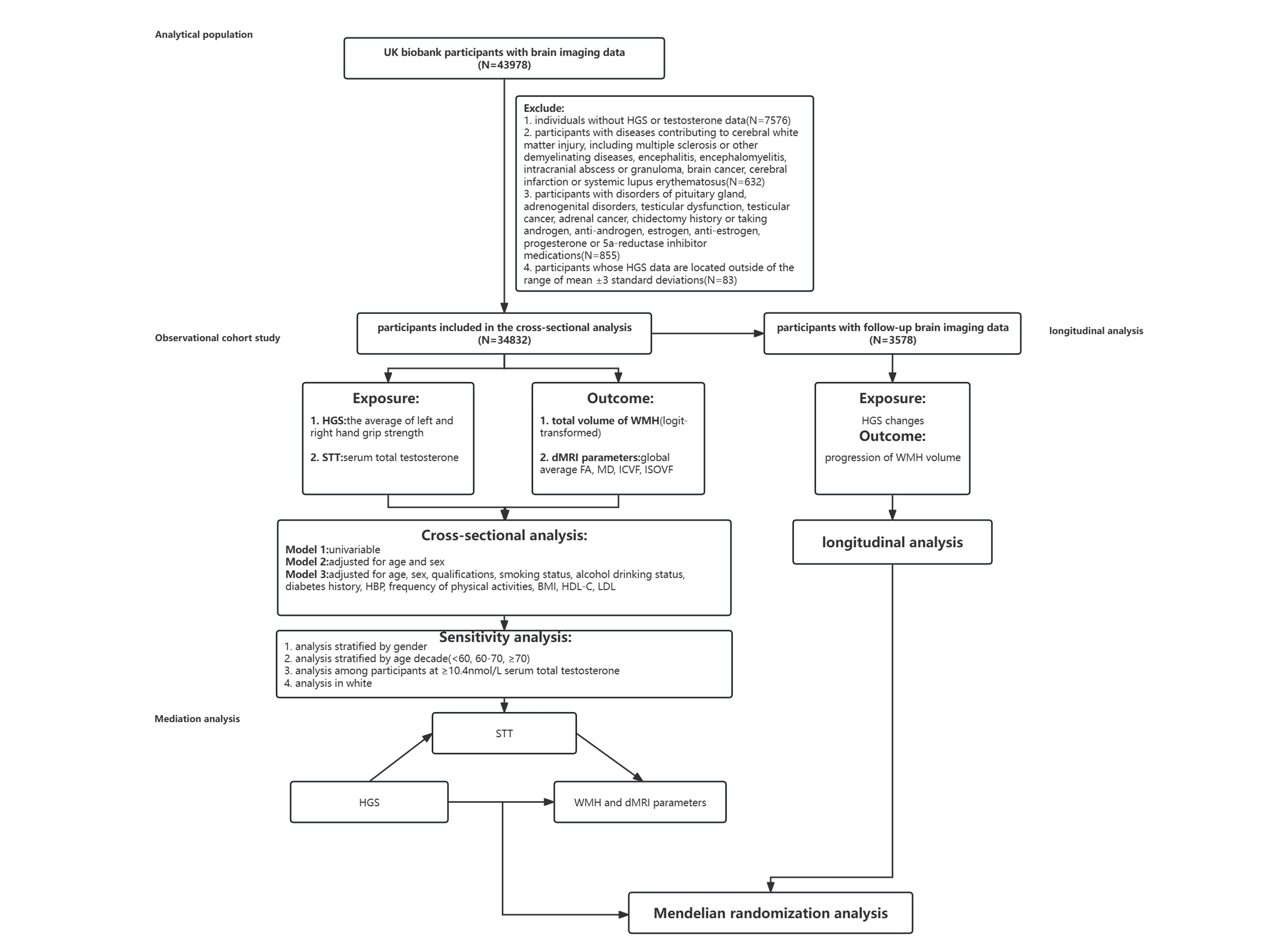


**Abbreviations:** WMH, white matter hyperintensity; dMRI, diffusion magnetic resonance imaging; FA, fractional anisotropy; MD, mean diffusivity; ICVF, intracellular volume fraction; ISOVF, isotropic volume fraction; HBP, high blood pressure; BMI, body mass index; HDL-C, high-density lipoprotein cholesterol; LDL, low-density lipoprotein

**Table S3. The basic information of genome-wide association study (GWAS) statistics.**

| **trait** | **year** | **sex** | **Sample size** | **Independent**  **SNPs number** |
| --- | --- | --- | --- | --- |
| Total Testosterone | 2020 | Males | 199569 | 44 |
| Hand grip strength (left) | 2018 | Males and Females | 461026 | 76 |
| IDP T2 FLAIR BIANCA WMH volume | 2018 | Males | 7705 | NA |
| Total testosterone levels | 2020 | NA | 194453 | NA |

**Abbreviations:** SNP, single nucleotide polymorphism; IDP, image-derived phenotype; BIANCA, brain intensity abnormality classification algorithm; FLAIR, fluid attenuated inversion recovery

**Figure S2.** **Associations between handgrip strength and neuroimaging outcomes stratified by age decade.**

**
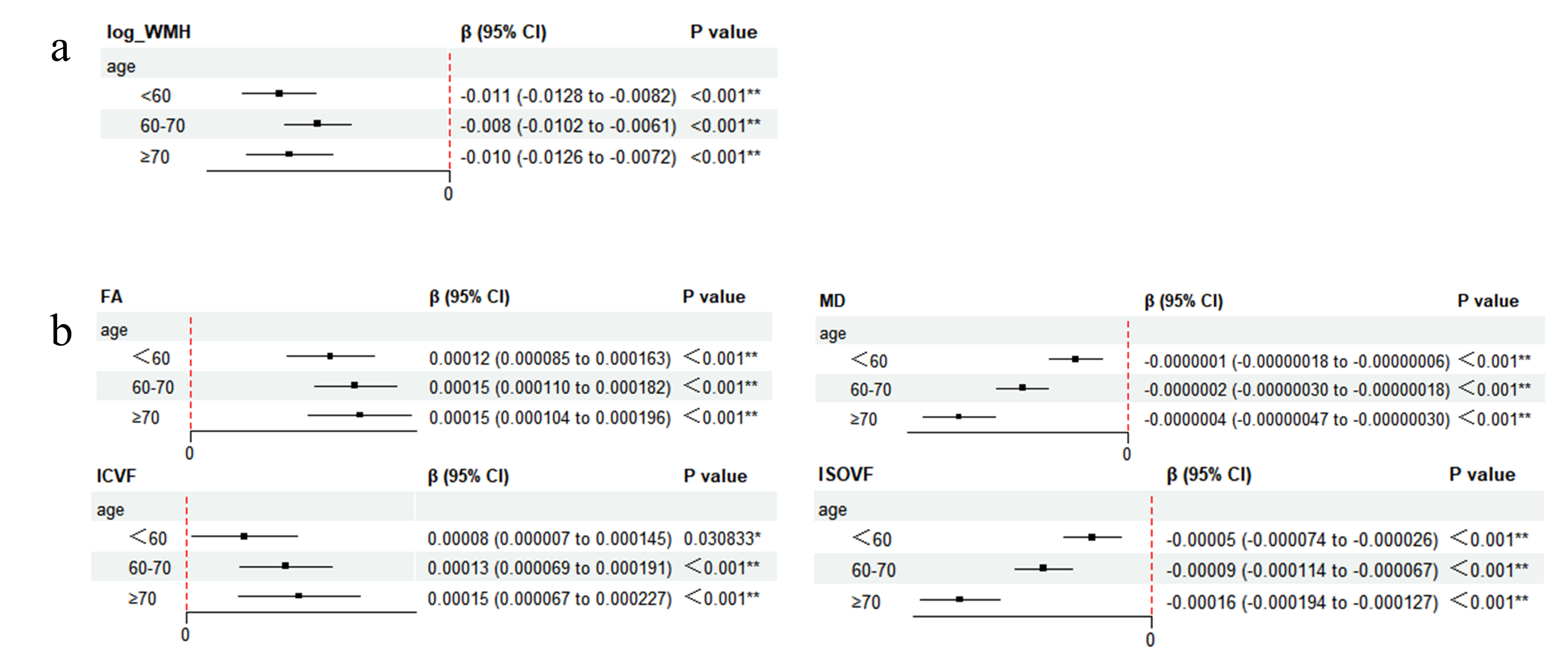
**

a. Associations between handgrip strength and white matter hyperintensity (WMH) stratified by age decade, log_WMH, logit-transformed value of white matter hyperintensity volume.

b. Associations between handgrip strength and white matter microstructural injury stratified by age decade, FA, fractional anisotropy; MD, mean diffusivity; ICVF, intracellular volume fraction; ISOVF, isotropic volume fraction.

**Figure S3. Associations between** **serum total testosterone and neuroimaging outcomes stratified by age decade.**

**
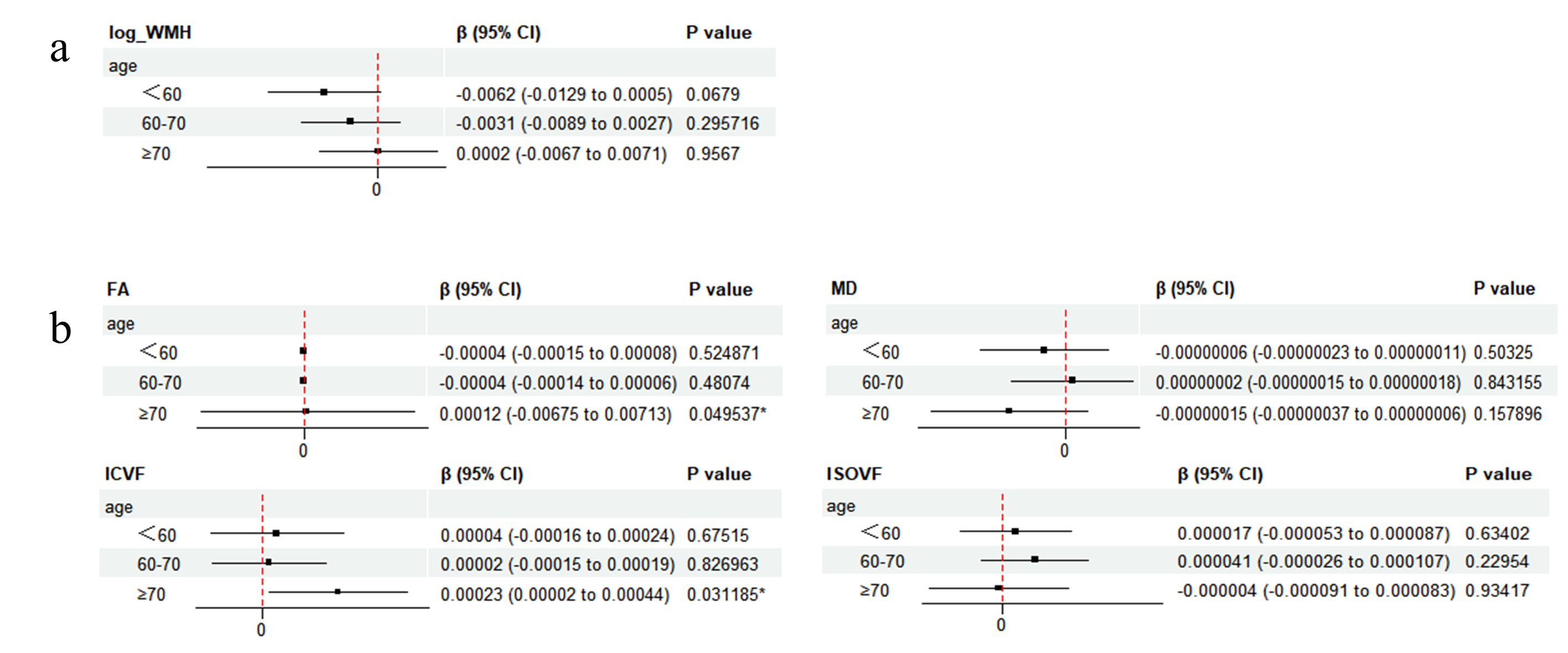
**

a. Associations between serum total testosterone and white matter hyperintensity (WMH) stratified by age decade, log_WMH, logit-transformed value of white matter hyperintensity volume.

b. Associations between serum total testosterone and white matter microstructural injury stratified by age decade, FA, fractional anisotropy; MD, mean diffusivity; ICVF, intracellular volume fraction; ISOVF, isotropic volume fraction.

**Table S4. Associations between serum total testosterone and neuroimaging outcomes among participants at ≥10.4nmol/L serum total testosterone.**

|  | **Model 1** | | | **Model 2** | | **Model 3** | |
| --- | --- | --- | --- | --- | --- | --- | --- |
| **Testosterone** | **β（95%CI）** | **P** | **β（95%CI）** | | **P** | **β（95%CI）** | **P** |
| **log_WMH** | -0.012892  (-0.01921466, -0.006568846) | <0.001 | -0.008185  (-0.01374876, -0.002621744) | | 0.00394 | 0.0006207  (-0.004900328, 0.006141745) | 0.825584 |
| **FA** | 8.862e-07  (-9.770256e-05, 0.0000994749) | 0.986 | -3.288e-05  (-1.291379e-04,6.337101e-05) | | 0.503 | -4.330e-05  (-1.403913e-04, 5.380033e-05) | 0.38211 |
| **MD** | -7.478e-08  (-2.469866e-07, 9.743024e-08) | 0.395 | 3.279e-08  (-1.251102e-07, 1.906882e-07) | | 0.684 | 3.087e-08  (-1.282753e-07, 1.900118e-07) | 0.703802 |
| **ICVF** | 8.482e-05  (-8.529775e-05, 0.0002549462) | 0.328 | 1.536e-05  (-1.489607e-04, 0.0001796816) | | 0.855 | 4.847e-05  (-1.174775e-04, 2.144203e-04) | 0.56697 |
| **ISOVF** | -1.711e-05  (-8.254703e-05, 4.831733e-05) | 0.608 | 1.659e-05  (-4.517346e-05, 7.835337e-05) | | 0.599 | 3.976e-05  (-2.258986e-05, 1.021078e-04) | 0.211336 |

Model 1 was not adjusted.

Model 2 was adjusted for sex and age.

Model 3 was adjusted for sex, age, qualifications, smoking status, alcohol drinking status, diabetes history, HBP, frequency of physical activities, BMI, HDL-C, LDL.

(**Abbreviations**: log_WMH, logit-transformed value of white matter hyperintensity volume; FA, fractional anisotropy; MD, mean diffusivity; ICVF, intracellular volume fraction; ISOVF, isotropic volume fraction)

**Table S5. Associations between handgrip strength and neuroimaging outcomes in the White.**

|  | **Model 1** | | **Model 2** | | **Model 3** | |
| --- | --- | --- | --- | --- | --- | --- |
| **HGS** | **β（95%CI）** | **P** | **β（95%CI）** | **P** | **β（95%CI）** | **P** |
| **log_WMH** | -0.0066766  (-0.007707668, -0.005645502) | <0.001 | -0.0056049  (-0.006934396, -0.004275305) | <0.001 | -0.0053187  (-0.0066338269, -0.004003576) | <0.001 |
| **FA** | 2.662e-04  (0.0002506296, 0.000281734) | <0.001 | 1.206e-04  (9.775215e-05, 0.0001434862) | <0.001 | 1.144e-04  (9.149844e-05, 1.372801e-04) | <0.001 |
| **MD** | -1.608e-07  (-1.881960e-07, -1.333428e-07) | <0.001 | -1.828e-07  (-2.208387e-07, -1.448567e-07) | <0.001 | -1.752e-07  (-2.131283e-07, -1.371681e-07) | <0.001 |
| **ICVF** | 1.808e-04  (0.0001534702, 0.0002080274) | <0.001 | 7.616e-05  (3.644599e-05, 0.0001158782) | <0.001 | 6.843e-05  (2.865346e-05, 0.0001082131) | <0.001 |
| **ISOVF** | -3.059e-05  (-4.136433e-05, -1.981798e-05) | <0.001 | -8.007e-05  (-9.53999e-05, -6.473188e-05) | <0.001 | -7.854e-05  (-9.390887e-05, -6.317602e-05) | <0.001 |

Model 1 was not adjusted.

Model 2 was adjusted for sex and age.

Model 3 was adjusted for sex, age, qualifications, smoking status, alcohol drinking status, diabetes history, HBP, frequency of physical activities, BMI, HDL-C, LDL.

(**Abbreviations**: HGS, handgrip strength; log_WMH, logit-transformed value of white matter hyperintensity volume; FA, fractional anisotropy; MD, mean diffusivity; ICVF, intracellular volume fraction; ISOVF, isotropic volume fraction)

**Table S6. Associations between serum total testosterone and neuroimaging outcomes in the White.**

|  | **Model 1** | | **Model 2** | | **Model 3** | |
| --- | --- | --- | --- | --- | --- | --- |
| **Testosterone** | **β（95%CI）** | **P** | **β（95%CI）** | **P** | **β（95%CI）** | **P** |
| **log_WMH** | 0.0125810  (0.0108333, 0.01432875) | <0.001 | -0.009228  (-0.01281034, -0.005644864) | <0.001 | -0.0005693  (-0.004154577, 0.003015953) | 0.755622 |
| **FA** | 2.983e-04  (0.0002716881, 0.0003249331) | <0.001 | 3.790e-05  (-2.378055e-05, 9.957361e-05) | 0.228 | 1.059e-05  (-5.184527e-05, 7.302460e-05) | 0.739556 |
| **MD** | 2.911e-07  (2.446383e-07, 3.376428e-07) | <0.001 | -2.633e-08  (-1.287761e-07, 7.611246e-08) | 0.614 | -1.597e-08  (-1.195363e-07, 8.760239e-08) | 0.76252 |
| **ICVF** | 9.477e-06  (-3.690229e-05, 5.585606e-05) | 0.689 | 5.162e-05  (-5.535314e-05, 0.0001586002) | 0.344 | 7.290e-05  (-3.546161e-05, 0.0001812619) | 0.187309 |
| **ISOVF** | 1.577e-04  (0.0001395158, 0.0001759197) | <0.001 | 8.966e-06  (-3.239246e-05, 5.032436e-05) | 0.671 | 3.417e-05  (-7.745058e-06, 7.608151e-05) | 0.110087 |

Model 1 was not adjusted.

Model 2 was adjusted for sex and age.

Model 3 was adjusted for sex, age, qualifications, smoking status, alcohol drinking status, diabetes history, HBP, frequency of physical activities, BMI, HDL-C, LDL.

(**Abbreviations**: log_WMH, logit-transformed value of white matter hyperintensity volume; FA, fractional anisotropy; MD, mean diffusivity; ICVF, intracellular volume fraction; ISOVF, isotropic volume fraction)

**Table S7.** **Characteristics of population in the cross-sectional and longitudinal analyses.**

|  | **Population in analyses** | | |
| --- | --- | --- | --- |
| **Characteristics** | **cross-sectional analysis** | **Longitudinal analysis** | **P** |
|  | N=34915 | N=3578 |  |
| **HGS (kg)** | 30.39 (10.38) | 31.45 (10.29) | <0.001 |
| **Testosterone(nmol/L)** | 6.79 (6.12) | 6.76 (6.18) | 0.785 |
| **sex = MALE** | 17820 (51.0%) | 1787 (49.9%) | 0.219 |
| **Age (years)** | 64.14 (7.75) | 61.41 (7.36) | <0.001 |
| **Qualifications** |  |  | 0.007 |
| unknown | 328 (0.9%) | 18 (0.5%) |  |
| None | 13470 (38.6%) | 1326 (37.1%) |  |
| College/university | 16897 (48.4%) | 1766 (49.4%) |  |
| A/As levels | 4220 (12.1%) | 468 (13.1%) |  |
| **Smoking** |  |  | 0.003 |
| unknown | 315 (0.9%) | 21 (0.6%) |  |
| No | 21737 (62.3%) | 2320 (64.8%) |  |
| Yes | 12863 (36.8%) | 1237 (34.6%) |  |
| **Alcohol** |  |  | 0.058 |
| unknown | 217 (0.6%) | 12 (0.3%) |  |
| No | 1058 (3.0%) | 97 (2.7%) |  |
| Yes | 33640 (96.3%) | 3469 (97.0%) |  |
| **Activity (days)** | 4.03 (2.17) | 3.98 (2.13) | 0.211 |
| **HBP** |  |  | <0.001 |
| unknown | 13032 (37.3%) | 1707 (47.7%) |  |
| No | 5276 (15.1%) | 534 (14.9%) |  |
| Yes | 16607 (47.6%) | 1337 (37.4%) |  |
| **BMI (kg/m^2^)** | 26.60 (4.35) | 26.42 (4.26) | 0.017 |
| **Diabetes** |  |  | 0.006 |
| unknown | 297 (0.9%) | 16 (0.4%) |  |
| No | 32787 (93.9%) | 3400 (95.0%) |  |
| Yes | 1831 (5.2%) | 162 (4.5%) |  |
| **HDL-C (mmol/L)** | 1.46 (0.35) | 1.47 (0.34) | 0.713 |
| **LDL (mmol/L)** | 3.58 (0.83) | 3.58 (0.83) | 0.849 |
| **log_WMH** | 8.01 (1.00) | 7.89 (0.96) | <0.001 |
| **FA (AU)** | 0.44 (0.02) | 0.44 (0.01) | <0.001 |
| **MD (AU)** | 0.00 (0.00) | 0.00 (0.00) | <0.001 |
| **ICVF (AU)** | 0.56 (0.03) | 0.56 (0.02) | <0.001 |
| **ISOVF (AU)** | 0.08 (0.01) | 0.08 (0.01) | <0.001 |

**Abbreviations:** HGS, hand grip strength; HBP, high blood pressure; BMI, body mass index; HDL-C, high-density lipoprotein cholesterol; LDL, low-density lipoprotein; log_WMH, logit-transformed value of white matter hyperintensity volume; FA, fractional anisotropy; MD, mean diffusivity; ICVF, intracellular volume fraction; ISOVF, isotropic volume fraction

**Figure S4. The mediating effects of serum total testosterone between handgrip strength and white matter hyperintensity.**


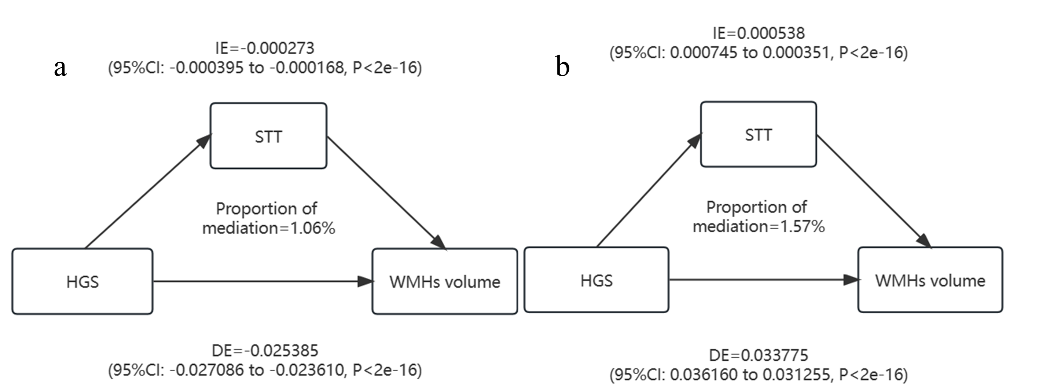


**a.** The mediating effects of serum total testosterone (STT) between handgrip strength (HGS) and white matter hyperintensity (WMH) volume in male.

**b.** The mediating effects of serum total testosterone (STT) between handgrip strength (HGS) and white matter hyperintensity (WMH) volume in female.

**Figure S5. The mediating effects of serum total testosterone between handgrip strength and white matter microstructural injury in female.**


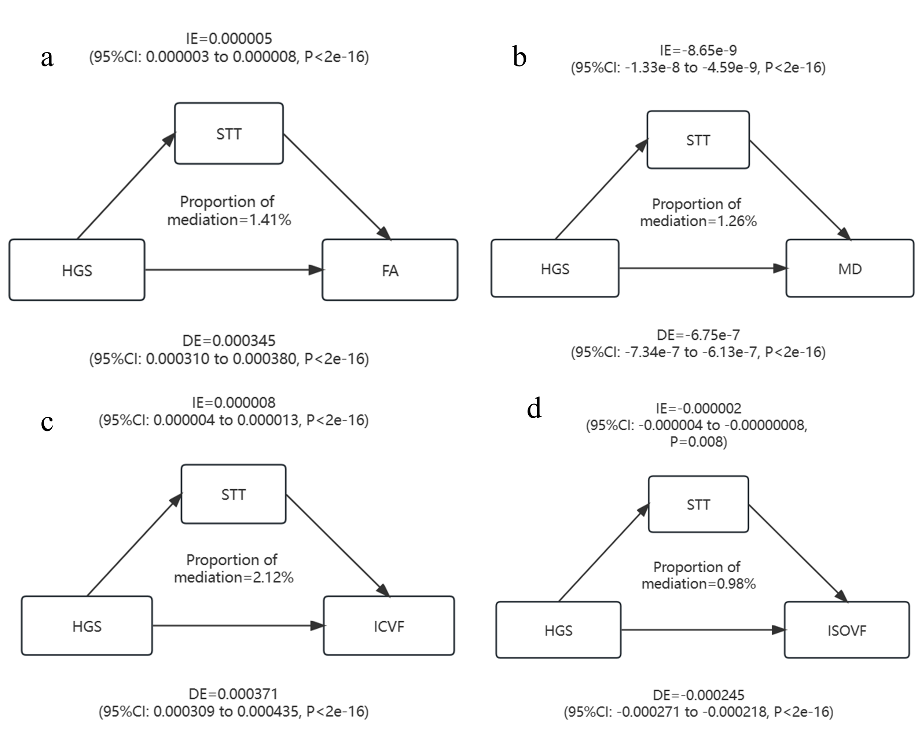


**a.** The mediating effects of serum total testosterone (STT) between handgrip strength (HGS) and fractional anisotropy (FA) in female.

**b.** The mediating effects of serum total testosterone (STT) between handgrip strength (HGS) and mean diffusivity (MD) in female.

**c.** The mediating effects of serum total testosterone (STT) between handgrip strength (HGS) and intracellular volume fraction (ICVF) in female.

**d.** The mediating effects of serum total testosterone (STT) between handgrip strength (HGS) and intracellular volume fraction (ISOVF) in female.

**Figure S6. Causal effects of handgrip strength (HGS) on serum total testosterone (STT).**

**
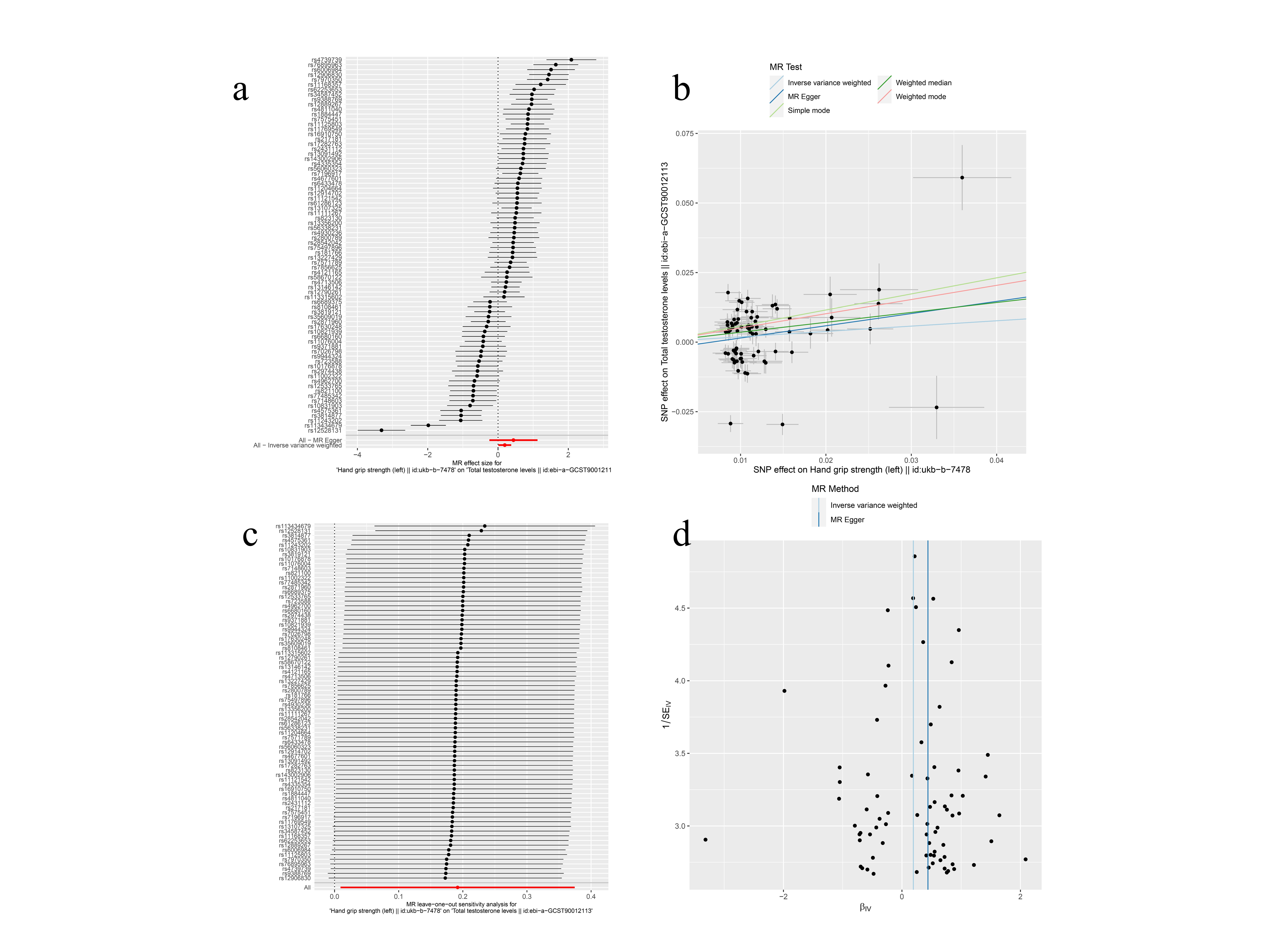
**

**a.** Forrest plot of SNPs associated with HGS and STT.

**b.** Scatter plot of SNPs associated with HGS and STT. The slopes of each line represent the causal association for each method.

**c.** Leave-one-out analysis of SNPs associated with HGS and STT.

**d.** Funnel plot of SNPs associated with HGS and STT.

**Figure S7. Causal effects of serum total testosterone (STT) on white matter hyperintensity (WMH).**

**
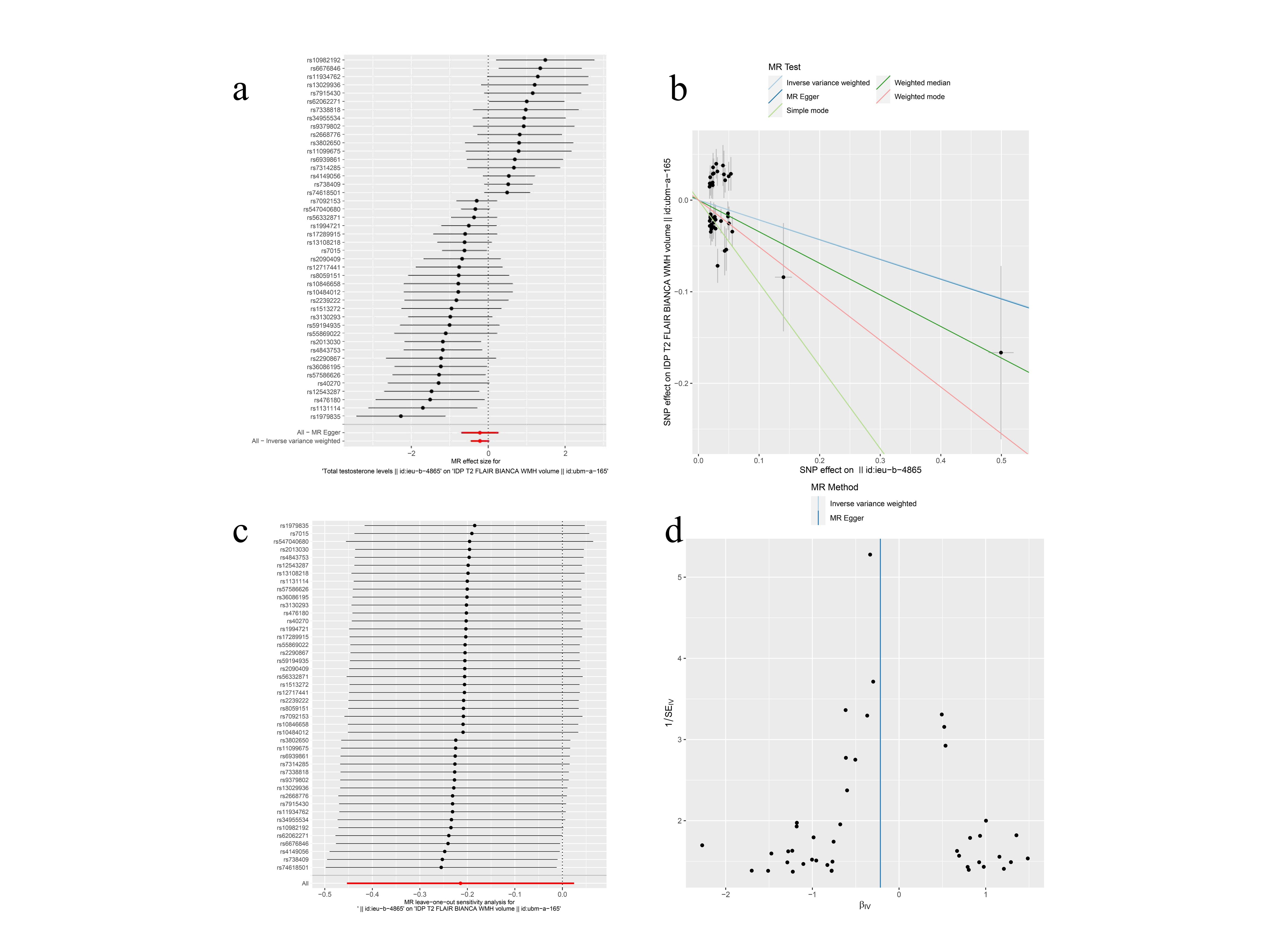
**

**a.** Forrest plot of SNPs associated with STT and WMH.

**b.** Scatter plot of SNPs associated with STT and WMH. The slopes of each line represent the causal association for each method.

**c.** Leave-one-out analysis of SNPs associated with STT and WMH.

**d.** Funnel plot of SNPs associated with STT and WMH.
